# Serological Screening for HIV, Hepatitis B, Hepatitis C, and Syphilis in Patients with Genital Molluscum Contagiosum: A Retrospective Cross-Sectional Study

**DOI:** 10.3390/v18090956

**Published:** 2026-09-01

**Authors:** Zuhal Erçin, Mehtap Toprak, Yasin Tiryaki

**Affiliations:** 1 Department of Dermatology, Sultan 2. Abdulhamid Han Training and Research Hospital, University of Health Sciences, 34668 Istanbul, Turkey; 2 Department of Pathology, Sultan 2. Abdulhamid Han Training and Research Hospital, University of Health Sciences, 34668 Istanbul, Turkey; dr.mehtapguner@hotmail.com; 3 Department of Medical Microbiology, Sultan 2. Abdulhamid Han Training and Research Hospital, University of Health Sciences, 34668 Istanbul, Turkey; yasintiryaki74@gmail.com

**Keywords:** genital molluscum contagiosum, serology, HIV, hepatitis B virus, hepatitis C virus, syphilis, sexually transmitted infections

## Abstract

(1) Background: The available literature on sexually transmitted infections in patients with genital molluscum contagiosum is limited. In this study, we aimed to evaluate the serological test results and screening rate for sexually transmitted infections, including human immunodeficiency virus, hepatitis B virus, hepatitis C virus, and syphilis, among patients with genital molluscum contagiosum. (2) Methods: In this retrospective cross-sectional study, we reviewed the medical records of patients aged ≥18 years diagnosed with genital molluscum contagiosum at our dermatology outpatient clinic between October 2016 and December 2025. Age, sex, and serological test results for anti-human immunodeficiency virus, hepatitis B surface antigen, anti-hepatitis C virus, and Venereal Disease Research Laboratory/Rapid Plasma Reagin were recorded. (3) Results: Serological screening was performed in 202 of 528 patients (38.3%), and 11 (5.45%) had positive test results for at least one sexually transmitted infection. (4) Conclusions: Our findings support routine screening for sexually transmitted infections in patients with genital molluscum contagiosum, given that a substantial proportion of these patients do not undergo serological screening.

## 1. Introduction

Molluscum contagiosum (MC) is a self-limiting, viral, infectious cutaneous disease caused by the molluscum contagiosum virus, a member of the Poxviridae family [1]. Molluscum contagiosum virus (MCV) is the only poxvirus for which humans are the sole natural hosts following the successful eradication of smallpox [2]. MC is a common infection worldwide, with an estimated prevalence of 5–11%, and its incidence appears to be increasing. Molluscum contagiosum virus DNA has been detected on clinically uninvolved skin and environmental fomites, suggesting that both direct and indirect contact may contribute to transmission [3]. The role of epidermal injury in the establishment of infection remains unclear [4]. Sexual or nonsexual direct contact with the infected skin, autoinoculation, and contaminated fomites are the main modes of transmission. Molluscum contagiosum virus is the only known member of the genus Molluscipoxvirus, and approximately 60% of its genes exhibit homology to those of variola and vaccinia viruses. MCV comprises four distinct genetic entities: MCV-1, which is responsible for most infections (76–97%), followed by MCV-2, whereas MCV-3 and MCV-4 are extremely rare. To date, complete genomes of only MCV-1 and MCV-2 have been deposited in GenBank (a genetic sequence database); therefore, current MCV subtyping assays can only reliably distinguish infections with these subtypes [2]. No clinical association has been identified between virus type and lesional morphology or anatomical distribution [4]. Patients who are immunocompromised, such as those with HIV (human immunodeficiency virus); who have undergone organ transplants; or who have cancer are more likely to contract MCV-2, which is responsible for roughly 60% of all molluscum contagiosum infections in immunocompromised populations [5]. Molluscum contagiosum is typically characterized by round umbilicated papules that vary in size and shape and range from pinkish to skin-colored [1]. Two main clinical patterns are recognized: lesions involving the face, neck, trunk, and arms, predominantly affecting children, and lesions involving the genitals, pubic region, lower abdomen, upper thighs, and buttocks, which are generally associated with sexual transmission [6]. Research on this prevalent skin condition has been relatively limited until recently [5]. In this study, we aimed to describe the demographic characteristics of patients with genital molluscum contagiosum and to evaluate the frequency and results of serological screening for selected sexually transmitted infections in a real-world dermatology outpatient setting.

## 2. Materials and Methods

### 2.1. Ethical Committee Approval

The study was conducted in accordance with the principles of the Declaration of Helsinki and was approved by the Ethics Committee of Istanbul Medipol University (protocol code: E-10840098-202.3.02-3556; approval date: 4 June 2025). As anonymized routine laboratory data were analyzed in this retrospective study, informed consent for participation was not required in accordance with the Turkish Regulation on Clinical Research, overseen by the Turkish Medicines and Medical Devices Agency, Ministry of Health.

### 2.2. Patient Cohort

A retrospective cross-sectional study was conducted based on a review of the outpatient records of all patients who visited the dermatology outpatient clinic of our hospital between October 2016 and December 2025. Outpatient clinic records were screened for molluscum contagiosum using the ICD-10 (International Classification of Diseases, 10th Revision) code B08.1. The medical records of patients aged ≥18 years who were diagnosed with molluscum contagiosum were retrospectively reviewed. As the ICD-10 code B08.1 includes both genital and extragenital molluscum contagiosum, only patients with genital involvement, confirmed through review of the clinical records, were included in the study. Demographic characteristics such as age, gender, and serological test results, including anti-human immunodeficiency virus (anti-HIV), anti-hepatitis C virus (anti-HCV), hepatitis B surface antigen (HBsAg), and Venereal Disease Research Laboratory/Rapid Plasma Reagin (VDRL/RPR) test results, were documented. In cases with reactive VDRL/RPR results, Treponema pallidum hemagglutination assay (TPHA) findings were also documented.

### 2.3. Serological Screening

HBsAg tests were performed using the Elecsys HBsAg II kit (Roche Diagnostics GmbH, Mannheim, Germany) on the Roche cobas e 801 system, employing the electrochemiluminescence immunoassay (ECLIA) method. Anti-HCV tests were performed on the Roche cobas e 801 system using the Elecsys Anti-HCV II kit (Roche Diagnostics GmbH, Mannheim, Germany) with the electrochemiluminescence immunoassay method. Syphilis serology was recorded as VDRL/RPR in the laboratory database and was performed using the Rapid Plasma Reagin (RPR) test with the Cromatest RPR Carbon kit (Linear Chemicals, S.L.U., Barcelona, Spain) by means of the macroscopic flocculation method, according to the manufacturer’s instructions. RPR-positive samples were confirmed using the Treponema pallidum hemagglutination assay with the DIALAB Syphilis TPHA kit (DIALAB GmbH, Vienna, Austria), according to the manufacturer’s instructions. Anti-HIV screening was performed using an electrochemiluminescence immunoassay (ECLIA) on the Roche cobas e 801 system with the Elecsys HIV Duo kit (Roche Diagnostics GmbH, Mannheim, Germany). Samples with reactive HIV screening results were confirmed using an HIV-1/2 antibody (Ab) differentiation immunoassay (Geenius HIV-1/2 Supplemental Assay, Bio-Rad Laboratories, Redmond, WA, USA).

### 2.4. Statistical Analysis

Statistical analyses were performed using IBM SPSS Statistics for Windows, Version 30.0 (IBM Corp., Armonk, NY, USA), with continuous variables presented as mean ± standard deviation (SD) and median values, and categorical variables presented as counts (*n*) and percentages (%). The normality of continuous variables was evaluated using the Shapiro–Wilk test and graphical methods (histograms and Q–Q plots), with comparisons between two groups performed using the Mann–Whitney U test. The prevalence of genital molluscum contagiosum, rates of serological testing, and seropositivity rates were calculated together with their 95% confidence intervals using the exact Clopper–Pearson binomial method. Differences in categorical variables were assessed using Fisher’s exact test. The association between sex and the rate of serological testing was assessed using relative risk (RR) with a 95% confidence interval (CI). Statistical significance was defined as a *p*-value < 0.05.

## 3. Results

### 3.1. Demographic Characteristics

**Figure 1:**
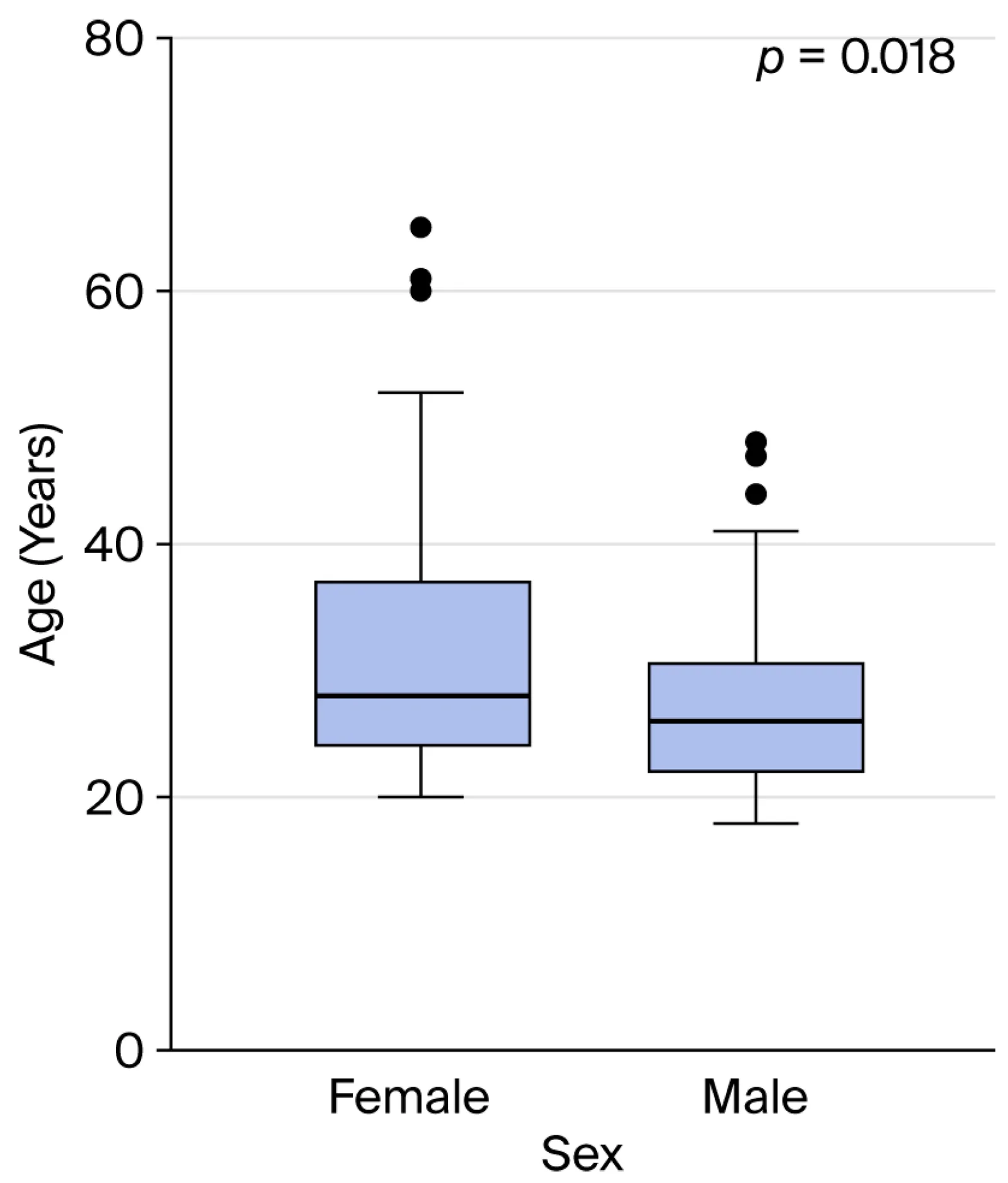
Distribution of age by gender among patients with genital molluscum contagiosum.

During the study period, a total of 389,721 patient visits were documented in the dermatology outpatient clinic. Of these, 528 cases of genital molluscum contagiosum were identified. Accordingly, the proportion of genital molluscum contagiosum among dermatology outpatient visits was 1.35‰ (95% CI: 1.24–1.48‰). Serological testing was requested in 202 of the 528 cases (38.3%). The 202 patients included in this study ranged in age from 18 to 65 years, with a mean age of 28.9 ± 8.9 years. Males accounted for 143 (70.8%) and females for 59 (29.2%) of the study population. The demographic and laboratory characteristics of patients with genital molluscum contagiosum are summarized in Table 1. Evaluation of age distribution demonstrated that female patients were significantly older than males, with mean ages of 31.7 ± 10.8 and 27.8 ± 7.8 years, respectively (*p* = 0.018) (Figure 1). No significant difference in age was detected between anti-HIV-positive and -negative patients (33.8 ± 11.8 vs. 28.8 ± 8.9 years; *p* = 0.370). Similarly, no significant association was observed between age and VDRL/RPR or HBsAg positivity (*p* = 0.927 and *p* = 0.769, respectively). Because anti-HCV positivity was observed in only a single patient, analyzing age distribution in this group was not feasible. Comparison of age between seropositive and seronegative patients revealed no statistically significant difference (30.6 ± 8.8 vs. 28.8 ± 9.0 years; *p* = 0.400), with age distribution by gender and serological status summarized in Table 2. In the cohort of 202 patients who underwent serological testing, anti-HIV positivity was observed in 3.4% of women (2/59) and 1.4% of men (2/143), with no statistically significant difference (*p* = 0.582). Anti-HCV positivity occurred in only one female patient (1.7%), with no cases observed in males (*p* = 0.292), and VDRL/RPR reactivity occurred solely in male patients (1.4%; 2/143), with no statistically significant difference observed between males and females (*p* = 0.999). Among male patients, the HBsAg positivity rate was 2.8% (4/143), with no cases detected among females (*p* = 0.324). Any type of serological test positivity was detected in 5.1% of female patients (3/59) and 5.6% of male patients (8/143), with no statistically significant difference between genders (*p* = 0.999).

### 3.2. Sexually Transmitted Infection Screening and Serological Findings

**Figure 2:**
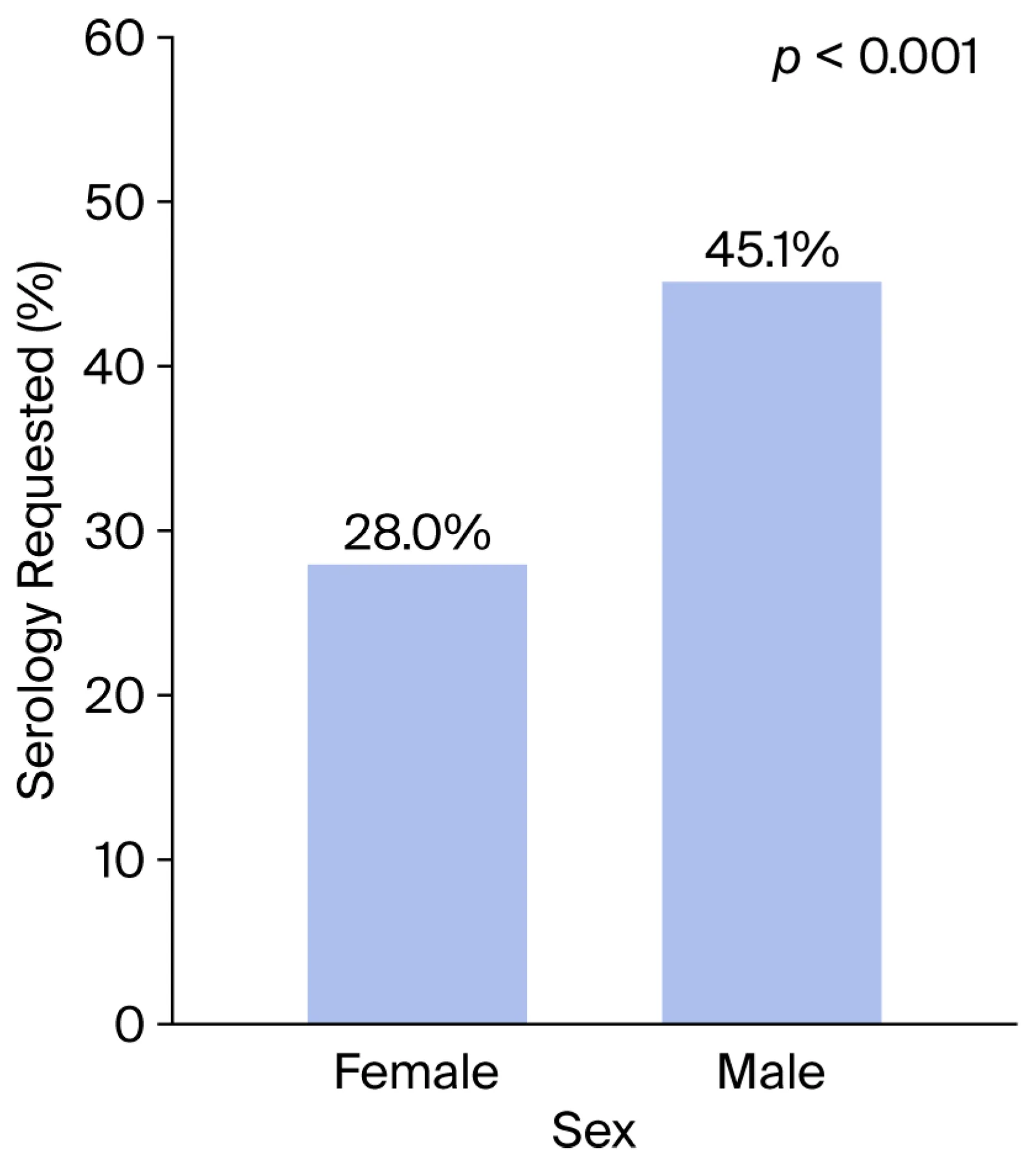
Serology testing rates by gender among patients with genital molluscum contagiosum.

Serological testing was requested in 202 of the 528 cases (38.3%), resulting in a serological testing rate of 45.1% (143/317) in men and 28.0% (59/211) in women; the likelihood of undergoing serological testing was significantly higher in men than in women (RR = 1.61; 95% CI: 1.25–2.08; *p* < 0.001) (Figure 2). Among the 202 patients with genital molluscum contagiosum who underwent serological testing, the anti-HIV seropositivity rate was 1.98% (4/202; 95% CI: 0.55–5.03%); the anti-HCV seropositivity rate was 0.50% (1/202; 95% CI: 0.01–2.75%); the VDRL/RPR reactivity rate was 0.99% (2/202; 95% CI: 0.12–3.54%) and all patients with reactive VDRL/RPR results also tested positive for TPHA; and the HBsAg positivity rate was 1.98% (4/202; 95% CI: 0.55–5.03%). Of the 202 patients who underwent serological testing, 11 had at least one positive serological result, corresponding to an overall seropositivity rate of 5.45% (95% CI: 2.75–9.53%). None of these 11 patients had positive results for more than one serological test concurrently. All four HBsAg-positive patients were male, aged 22, 23, 27, and 35 years. The only anti-HCV-positive patient was a 40-year-old woman. Both patients with positive syphilis serology were men, aged 23 and 32 years. Among the four HIV-positive patients, two were men aged 20 and 47 years with chronic asymptomatic infection, whereas the two women, aged 29 and 39 years, were classified as late presenters.

## 4. Discussion

The serological profile of sexually transmitted infections has been examined in patients with condyloma acuminata in the literature [7,8]. However, data regarding these infections in patients with genital molluscum contagiosum remain limited, as there have been no studies specifically evaluating this issue in patients with this condition. In this study, we aimed to evaluate the serological test results and screening rate for sexually transmitted infections, including human immunodeficiency virus, hepatitis B virus, hepatitis C virus, and syphilis, among patients with genital molluscum contagiosum.

In our study, the prevalence of genital molluscum contagiosum among patients attending the outpatient clinic was 1.35 per 1000 patients. Based on the results of a study conducted in a sexually transmitted infections unit, molluscum contagiosum cases accounted for 1% of patients [9]. To the best of our knowledge, no studies have investigated genital molluscum contagiosum prevalence in dermatology outpatient clinics, nor has the serological testing rate in patients with genital molluscum contagiosum been reported. In our study, serological testing was requested for 202 (38.3%) of the 528 total genital molluscum contagiosum cases, and specifically in 45.1% of male and 28% of female patients, demonstrating a significantly higher likelihood of testing in men. Male patients are more likely to engage in high-risk behaviors [10], which may have prompted clinicians to request serological testing more often in this group. In our study, the anti-HIV seropositivity rate was 1.98%, which is lower than the 6.5% coinfection rate reported in a study conducted in a sexually transmitted infections unit [9]. This lower rate may be explained by the fact that our study was conducted in a general dermatology outpatient clinic rather than in a sexually transmitted infection unit. The anti-HCV seropositivity rate in our study was 0.50%, representing the lowest seropositivity rate among the infections evaluated, which may be due to the highly inefficient sexual transmission of HCV (hepatitis C virus) [11]. In a previous study, the maximum HCV transmission incidence through sexual contact was reported to be 0.07% per year, or approximately 1 per 190,000 sexual contacts [12]. The HBsAg seropositivity rate in our study was 1.98%, identical to the anti-HIV seropositivity rate, which may be explained by the fact that HBV (hepatitis B virus) and HIV share major modes of transmission [13]. HBV is a highly resilient organism; in chronically infected individuals, it is present at a high concentration in the blood but is also found in vaginal secretions, semen, breast milk, urine, and tears [14]. HBV can also be transmitted vertically, with mother-to-child transmission representing an important contributor to the global burden of chronic HBV infection [15]. In our study, serological evidence of syphilis was detected in 0.99% of patients. In a 1991 study on patients with genital molluscum contagiosum, syphilis was detected similarly in 0.46% of cases; however, HIV, HBV, and HCV were not included in the screening [16]. In our study, age distribution analysis demonstrated that the mean age of female patients was significantly higher than that of male patients (*p* = 0.018). In a previous study on trends in molluscum contagiosum in the United States, women with the infection were found to be younger than their male counterparts [17], a finding which contrasts with the results of our study. This finding may be attributable to the greater likelihood of younger women with genital molluscum contagiosum presenting to obstetrics and gynecology clinics, whereas older women may be more likely to seek care in dermatology clinics in our country. The mean age of the study population was 28.9 ± 8.9 years, results similar to those of a study performed in a sexually transmitted infections unit, in which the mean patient age was 29.7 years [9].

The presence of one sexually transmitted infection is well recognized as a risk factor for subsequent acquisition of other sexually transmitted infections [7]. In sexually transmitted cases, molluscum contagiosum predominantly affects the anogenital region, including the external genitalia, inguinal folds, inner thighs, and suprapubic area. The 2020 European guideline on the management of genital molluscum contagiosum recommends screening for other sexually transmitted infections in patients presenting with genital molluscum contagiosum [6]. In this retrospective cohort of 528 patients with genital molluscum contagiosum, only 38.3% underwent serological screening for HIV, hepatitis B, hepatitis C, and syphilis. Among those screened, 5.45% had serological evidence of at least one infection. Although the overall number of seropositive patients was limited, the detection of HIV, hepatitis B, hepatitis C, and syphilis among screened patients underscores the importance of appropriate sexually transmitted infection assessment in patients presenting with genital molluscum contagiosum. These findings highlight a substantial gap between recommended sexually transmitted infection assessment and real-world screening practices in patients presenting with genital molluscum contagiosum. The under-screening of patients with this condition for concomitant sexually transmitted infections has important public health implications and highlights the need for greater physician awareness. The limitations of this study include its single-center design and relatively small sample size. In addition, the lack of available data on screening for Chlamydia trachomatis and Neisseria gonorrhoeae infections limited our ability to assess the prevalence of these infections in our study population. Nevertheless, our study provides valuable real-world insights and emphasizes the need to improve awareness of this issue among physicians managing patients with genital molluscum contagiosum.

## 5. Conclusions

Molluscum contagiosum is a self-limiting, viral, infectious cutaneous disease caused by the molluscum contagiosum virus, a member of the Poxviridae family [1]. Two main clinical presentations are observed: lesions on the face, neck, trunk, and arms, seen predominantly in children, and on the genitals, pubic region, lower abdomen, upper thighs, and buttocks, which appear to be associated with sexual transmission [6]. The serological profile of sexually transmitted infections has been examined in patients with condyloma acuminata in the literature [7,8]. However, data regarding these infections in patients with genital molluscum contagiosum remain limited, as there have been no studies specifically evaluating this issue in patients with this condition. The 2020 European guideline on the management of genital molluscum contagiosum recommends that patients presenting with the disease be offered screening for other sexually transmitted infections [6]. The results obtained in our study lend support to this recommendation and also reveal that a proportion of patients with genital molluscum contagiosum do not undergo screening for concomitant sexually transmitted infections. The under-screening of patients with genital molluscum contagiosum for concomitant sexually transmitted infections has important public health implications and highlights the need for increased awareness among physicians managing these patients.

## Figures and Tables

**Table 1:** Demographic and laboratory characteristics of patients diagnosed with genital molluscum contagiosum.

	*N* = 202
Age (years), mean ± standard deviation	28.9 ± 8.9
Male gender, *n* (%)	143 (70.8)
Female gender, *n* (%)	59 (29.2)
Anti-HIV, *n* (%)	4 (1.98)
Anti-HCV, *n* (%)	1 (0.50)
VDRL/RPR, *n* (%)	2 (0.99)
HBsAg, *n* (%)	4 (1.98)
Any positive serology, *n* (%)	11 (5.45)

**Table 2:** Age distribution of gender and serological groups.

		Age (Years) Mean ± SD/Median	*p* *
Gender	Female	31.7 ± 10.8/28	0.018
	Male	27.8 ± 7.8/26	
Anti-HIV	Negative	28.8 ± 8.9/27	0.370
	Positive	33.8 ± 11.8/34	
VDRL/RPR	Negative	28.9 ± 9.0/27	0.927
	Positive	27.5 ± 6.4/27.5	
HBsAg	Negative	28.9 ± 9.0/27	0.769
	Positive	26.8 ± 5.9/25	
Any positive serology	Negative	28.8 ± 9.0/27	0.400
	Positive	30.6 ± 8.8/29	

* Mann–Whitney U test.

## Data Availability

The data in this study are available upon request from the corresponding author due to privacy restrictions.

## Abbreviations

The following abbreviations are used in this manuscript:

Ab	Antibody
Anti-HCV	Anti-hepatitis C virus
Anti-HIV	Anti-human immunodeficiency virus
CI	Confidence interval
ECLIA	Electrochemiluminescence immunoassay
GenBank	Genetic sequence databank
HbsAg	Hepatitis B surface antigen
HBV	Hepatitis B virus
HCV	Hepatitis C virus
H&E	Hematoxylin and eosin
HIV	Human immunodeficiency virus
ICD-10	International Classification of Diseases-10th Revision
MC	Molluscum contagiosum
MCV	Molluscum contagiosum virus
RPR	Rapid Plasma Reagin
RR	Relative risk
SD	Standard deviation
TPHA	Treponema pallidum hemagglutination assay
VDRL/RPR	Venereal Disease Research Laboratory/Rapid Plasma Reagin
